# Access to kidney transplantation in Mexico, 2007–2019: a call to end disparities in transplant care

**DOI:** 10.1186/s12882-021-02294-1

**Published:** 2021-03-19

**Authors:** Guillermo Garcia-Garcia, Marcello Tonelli, Margarita Ibarra-Hernandez, Jonathan S. Chavez-Iñiguez, Ma. Concepcion Oseguera-Vizcaino

**Affiliations:** 1 Nephrology Service, Hospital Civil de Guadalajara Fray Antonio Alcalde, Centro Universitario de Ciencias de la Salud, Universidad de Guadalajara, Hospital 278, Jal., CP 44280 Guadalajara, Mexico; 2 Department of Medicine, University of Calgary, A100, Administration Building, 2500 University Drive NW, Calgary, AB T2N 1N4 Canada

**Keywords:** Kidney transplantation, Disparities, End-stage kidney disease, Organ donation

## Abstract

**Background:**

Access to kidney transplantation is limited to more than half of the Mexican population.

A fragmented health system, gender, and sociocultural factors are barriers to transplant care. We analyzed kidney transplantation in Mexico and describe how public policies and sociocultural factors result in these inequities.

**Methods:**

Kidney transplant data between 2007 to 2019 were obtained from the National Transplant Center database. Transplant rates and time spent on the waiting list, by age, gender, health system, and insurance status, were estimated.

**Results:**

During the study period 34,931 transplants were performed. Recipients median age was 29 (IQR 22–42) years, 62.4% were males, and 73.9% were insured. 72.7% transplants were from living-donors. Annual transplant rates increased from 18.9 per million population (pmp) to 23.3 pmp. However, the transplant rate among the uninsured population remained low, at 9.3 transplants pmp. In 2019, 15,890 patients were in the waiting list; 60.6% were males and 88% were insured. Waiting time to transplant was 1.55 (IQR 0.56–3.14) years and it was shorter for patients listed in the Ministry of Health and private facilities, where wait lists are smaller, and for males. Deceased-organ donation rates increased modestly from 2.5 pmp to 3.9 pmp.

**Conclusions:**

In conclusion, access to kidney transplantation in Mexico is unequal and restricted to patients with medical insurance. An inefficient organ procurement program results in low rates of deceased-donor kidneys. The implementation of a comprehensive kidney care program, recognizing kidney transplantation as the therapy of choice for renal failure, offers an opportunity to correct these inequalities.

## Background

*“If not us, then who?If not now, then when?”*

*John Lewis.*

Kidney transplantation has been advocated as the preferred modality of treatment for eligible patients with chronic kidney disease (CKD), including children, in terms of cost-effectiveness, better quality of life, and long-term survival [1, 2].

The first kidney transplant (living donor) in Mexico was performed in 1963. In 1984 legislation on organ and tissue donation and transplantation was passed by the Mexican congress, followed by the creation of the National Transplant Registry in 1988, to promote and coordinate tissue and organ transplantation in the country [3]. In 1999, the transplant registry was replaced by a central coordinating center, the National Transplant Center (CENATRA by its Spanish acronym), and by the establishment of a network of nationwide transplant coordinating centers [4, 5]. These changes resulted in an impressive increase in kidney transplantation in Mexico. Between 1984 and 2019, the transplant rate increased from 1.57 per million population (pmp) to 23.2 pmp, and the proportion of deceased-organ donor kidney transplantation increased from 12 to 31% [6].

However, because of the fragmentation of Mexico’s health system, access to kidney transplantation among the uninsured population remains limited to patients who could afford the cost of transplantation and maintaining immunosuppressive therapy [7]. Similarly, deceased-organ retrieval and allocation is also fragmented, resulting in an unequal and inefficient deceased-donor organ procurement and allocation [4]. Additionally, the Mexican health care system is complex and highly bureaucratic, which limits the access to kidney transplantation, especially for vulnerable populations [8, 9]. The result is a low transplantation rate among patients without health care insurance (13 pmp) in comparison to the national rate (24 pmp) [4, 10]. The rates of deceased organ donation (3.2 pmp) and deceased-donor organ kidney transplantation (7.2 pmp) are among the lowest in Latin America [11]. Also, gender disparities have been described, with more than 60% of transplant recipients being males [8].

In this report, we describe the inequities in organ donation and access to kidney transplantation in Mexico, and how current public policies and sociocultural factors may influence or exacerbate these inequities.

## Methods

Kidney transplant data from 2007 to 2019 were obtained from the National Transplant Center’s (CENATRA by its Spanish acronym) kidney wait list file [12], kidney transplant files [13], and organ and tissue donor files [14] . Patients with missing information and those < 3 years old were excluded. Age at transplant, gender, health system of transplant, insurance status, date of transplant; date of placement in the waiting list (deceased-donor only), time spent on the waiting list until transplanted, state where the transplant was performed; donor type (deceased, living-related, living-unrelated), health system where deceased-donor kidneys were retrieved, and state of organ procurement were recorded. Transplant rates were estimated per million population, using population census data from the National Institute of Statistics, Geography and Informatics (INEGI) [15], and population estimates by the Consejo Nacional de Población (CONAPO by its Spanish acronym) [16]. Due to the lack of a national dialysis registry, time spent on the waiting list was estimated from the date of listing to the date of transplant. Transplant counts and unadjusted rates by age group, gender, and insurance status are presented on annual basis. Insurance status was estimated from INEGI data [15]. No data was available to estimate patient and graft survival. Categorical variables are presented by frequency distributions; age, and time on the waiting list, are presented as median and interquartile range (IQR). For comparisons between groups, chi-squared test and the Mann-Whitney test were used when appropriate. Statistical analysis was done using the SPSS software, version 21.0 ((SPSS Inc., Chicago IL).

## Results

### Kidney transplantation

Between 2007 and 2019, 35,107 transplants were performed; after excluding 126 patients with missing information and 50 patients < 3 years old, 34,931 transplants were included in the analysis. Median age among recipients was 29 (IQR 22–42) years; they were largely male (62.4%), and over 91.4% of the transplants were performed in the 15–64 age group; the majority (*n* = 34,919) were kidney-alone transplants whereas 12 were combined kidney-pancreas transplants.

Overall, 25,423 (72.7%) transplants were from living-donors (LD), and 9508 (27.2%) from brain death deceased-donors (DD); 3092 (8.9%) originated from living-unrelated donors (LURD). In 20 (0.1%) cases the organ origin was unknown. The largest proportion (73.9%) of transplant recipients were patients with health care insurance, and over half of all transplants were performed at the Mexican Institute of Social Security (IMSS by its Spanish acronym), the largest dialysis and kidney transplant provider in Mexico (Table 1). The counts and transplantation rates by age group, gender, insurance status, and organ origin are presented in Table 2.

**Table 1:** Sociodemographic and clinical characteristics of patients receiving a kidney transplant between 2007 and 2019

	n = 34,931
Age (y)	29 (IQ 22–42)
Age group (%)	
3–14	2230 (6.4)
15–29	16,022 (46.0)
30–64	15,904 (45.5)
≥ 65	775 (2.2)
Gender (%)	
Male	21,803 (62.4)
Female	13,128 (37.6)
Insurance status (%)	
Insured	25,825 (73.9)
Uninsured	9106 (26.1)
Organ origin (%)	
Living-related Donor	22,311 (64.0)
Living-unrelated Donor	3092 (9.0)
Deceased-Donor	9508 (27.2)
Unknown	20 (0.1)
Organs	
Kidney-alone	34,919 (99.9)
Pancreas-Kidney	12 (0.0)
Health System (%)	
IMSS	18,068 (52.0)
Ministry of Health	9106 (26.1)
Private	6126 (17.5)
ISSSTE	1088 (3.1)
Armed Forces	454 (1.3)
PEMEX	87 (0.2)

*IMSS* Mexican Institute for Social Security; *ISSSTE* Institute for Social Security of Civil Servants; *PEMEX* Petroleos Mexicanos

**Table 2:** Transplantation counts and unadjusted transplantation rates per million population (pmp), by age, gender, insurance status, and organ origin

Year	2007 (pmp)	2008 (pmp)	2009 (pmp)	2010 (pmp)	2011 (pmp)	2012 (pmp)	2013 (pmp)	2014 (pmp)	2015 (pmp)	2016 (pmp)	2017 (pmp)	2018 (pmp)	2019 (pmp)	total
Age (y)														
3–14	272 (10.1)	268 (9.9)	179 (6.6)	172 (6.3)	209 (7.7)	145 (5.3)	171 (6.3)	129 (4.8)	142 (5.2)	152 (5.6)	140 (5.2)	126 (4.7)	125 (4.6)	2230
15–29	863 (29.3)	1051(35.3)	1181 (39.1)	1253 (40.9)	1295 (41.8)	1290 (41.2)	1296 (41.0)	1263 (39.7)	1363 (42.5)	1410 (43.8)	1384 (42.8)	1234 (38.0)	1139 (35.0)	16,022
30–64	906 (22.9)	901 (22.2)	945 (22.7)	952 (22.3)	1060 (24.3)	1171 (26.2)	1241 (27.1)	1221 (26.1)	1268 (26.6)	1399 (28.7)	1558 (31.3)	1676 (33.1)	1606 (31.1)	15,904
≥ 65	38 (6.0)	36 (5.5)	45 (6.6)	40 (5.7)	42 (5.8)	64 (8.6)	58 (7.5)	52 (6.5)	71 (8.6)	74 (8.4)	83 (9.4)	74 (8.1)	98 (10.4)	775
Gender														
Male	1232 (23.1)	1365 (25.2)	1444 (26.3)	1507 (27.0)	1612 (28.5)	1697 (29.6)	1726 (29.7)	1662 (28.3)	1779 (29.9)	1912 (31.8)	1985 (32.6)	1979 (32.2)	1903 (30.7)	21,803
Female	847 (15.2)	891 (15.7)	906 (15.8)	910 (15.6)	994 (16.8)	973 (16.3)	1040 (17.2)	1003 (16.3)	1065 (17.1)	1123 (17.9)	1180 (18.6)	1131 (17.6)	1065 (16.4)	13,128
Insured														
yes	1524 (32.2)	1643 (34.2)	1751 (35.9)	1750 (35.4)	1882 (37.5)	1936 (38.1)	2038 (39.6)	1905 (36.5)	2148 (40.7)	2260 (42.3)	2357 (43.7)	2335 (42.8)	2296 (41.7)	25,825
No	555 (9.0)	613 (9.8)	599 (9.4)	667 (10.3)	724 (11.0)	734 (11.0)	728 (10.8)	760 (11.2)	696 (10.1)	775 (11.1)	808 (11.5)	775 (10.9)	672 (9.3)	9106
Organ														
DD	508 (4.6)	574 (5.1)	512 (4.5)	516 (4.5)	630 (5.4)	724 (6.1)	766 (6.4)	757 (6.3)	823 (6.7)	867 (7.0)	933 (7.5)	979 (7.8)	919 (7.2)	9508
L-RD	1454 (13.3)	1555 (14.0)	1687 (15.0)	1764 (15.5)	1815 (15.7)	1785 (15.2)	1814 (15.3)	1662 (13.8)	1699 (14.0)	1831 (14.9)	1868 (15.0)	1739 (13.8)	1638 (12.9)	22,311
L-URD	115 (1.0)	121 (1.0)	146 (1.3)	136 (1.1)	160 (1,3)	157 (1.3)	185 (1.5)	246 (2.0)	322 (2.6)	337 (2.7)	364 (2.9)	392 (3.1)	411 (3.2)	3092
Unknown	2	6	5	1	1	4	1	0	0	0	0	0	0	20
Total	2079 (18.9)	2256 (20.1)	2350 (20.8)	2417 (21.1)	2606 (22.4)	2670 (22.6)	2766 (23.2)	2665 (22.1)	2844 (23.3)	3035 (24.6)	3165 (25.4)	3110 (24.7)	2968 (23.3)	34,931

*pmp* per million population; *DD* deceased donor; *L-RD* living-related donor; *L-URD* living-unrelated donor

**Fig. 1:**
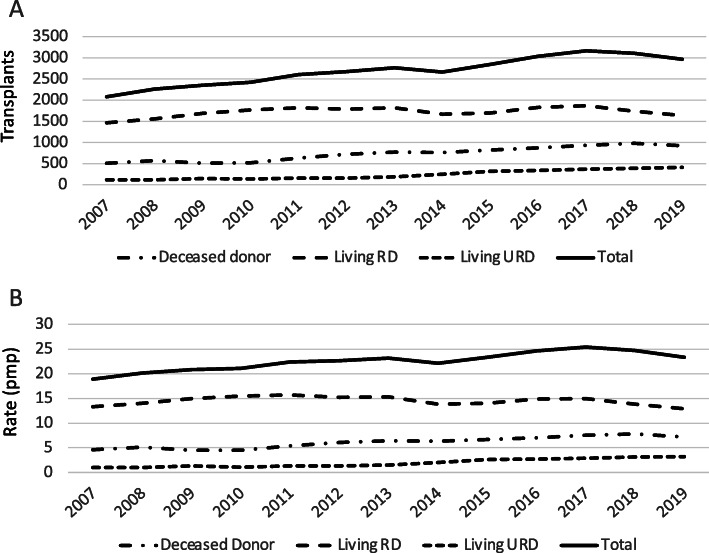
Number of kidney transplants (**a**), and unadjusted transplant rates pmp (**b**), by donor type 2007–2019

During the study period, the annual number of transplants increased 42.7%, from 2079 transplants in 2007 to 2968 transplants in 2019 (Fig. 1a); the number of DD transplants increased 80.9%, from 508 in 2007 to 919 in 2019, while the number of LD transplants increased 30.5%, from 1569 to 2049 (Table 2). Interestingly, the rate of LURD transplants had a three-fold increase in the same period (Table 2), although the absolute magnitude of the increase was relatively small.

**Fig. 2:**
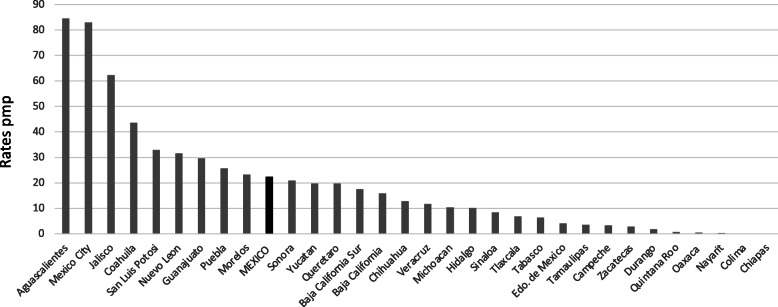
Unadjusted transplant rates per million population, by state

The annual transplant rate per million population (pmp) increased gradually from 18.9 pmp in 2007 to 23.3 pmp in 2019. The rise was driven mainly by the increase in the rate of DD transplants and the rate of LURD transplants (Fig. 1b). The largest transplant rates were reported in the states of Aguascalientes (84.5 pmp), Mexico City (82.9 pmp), Jalisco (62.3 pmp), and Coahuila (43.6 pmp) (Fig. 2).

**Fig. 3:**
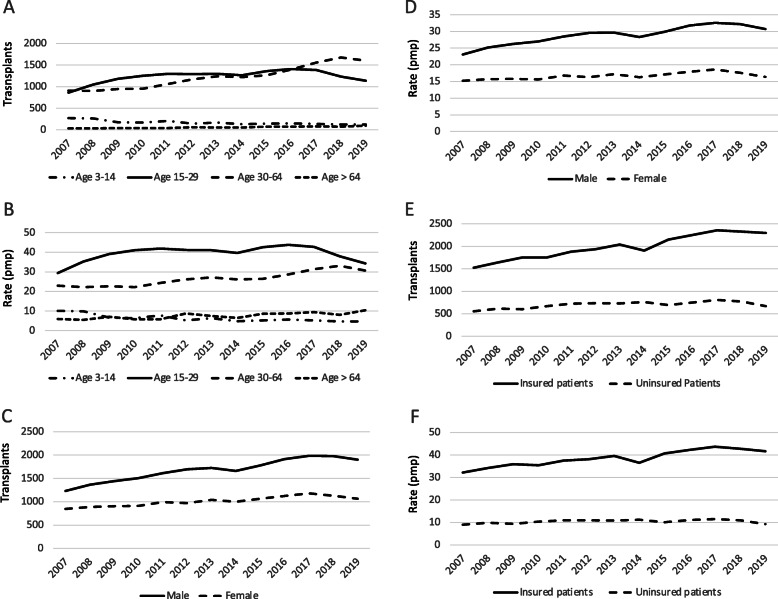
Number of kidney transplants and unadjusted transplant rates per million population, by recipient age (**a, b**), gender (**c, d**), and insurance status (**e,f**), 2007–2019

Overall, the proportion of transplants from DD increased from 24.4% in 2007 to 30.9% in 2019, while the proportion of transplants from LD decreased from 75.4 to 69.0%. The increase in the number (Fig. 3a) and the rate of transplantation was observed in all age groups, except in the 3–15 age group where an actual decline was observed (Fig. 3b). Males received more transplants than females. Although the number of transplants in females increased (Fig. 3c), the gap in transplant rates between males and females actually widened (Fig. 3d), and the proportion of transplants performed in women declined from 40.7% in 2007 to 35.8% in 2019. Transplant counts in uninsured patients increased modestly (Fig. 3e); however, the annual transplant rate among the uninsured population remained persistently low, at about 10.0 transplants pmp (Fig. 3f), and the proportion of transplants in this group declined from 27.6 to 23.2% in the study period.

Transplantation practices differed between insured and uninsured patients and between health systems. Insured patients were older (29 years (IQR 22–43) vs 27 years (IQR 20–40), *p* = 0–0001), and the proportion of recipients of DD kidney transplants was significantly lower (21.4% vs 43.9%, *p* = 0.0001), in comparison with the uninsured population. On the contrary, the proportion of transplants from LURD was two-fold higher (10.1% vs 5.3%, p = 0.0001) in patients with health care insurance in comparison with uninsured patients. This was especially remarkable in private transplant facilities, where LURD represented 20.2% of all the transplants performed in this setting, in comparison to 6.4% in public facilities (p = 0.0001).

#### Waiting list

On December 31, 2019, the cumulative kidney transplant waiting list had 17,081 candidates, of whom 16,487 patients were listed on the deceased-donor waiting list between 2007 and 2019. We excluded 34 patients from the analysis; in 20 cases because of missing information, and 15 cases because they were < 3 years old, leaving a total of 15,890 for the analysis. Their median age was 36 (IQR 27–49) years, and 9631 (60.6%) were males. Sixty-three percent were in the 30–64 age group. The largest proportion of listed patients belonged to IMSS patients (81.1%), followed by the Ministry of Health (12.4%), and the Institute for Social Security of Civil Servants (ISSSTE by its Spanish acronym) (3.4%).

**Fig. 4:**
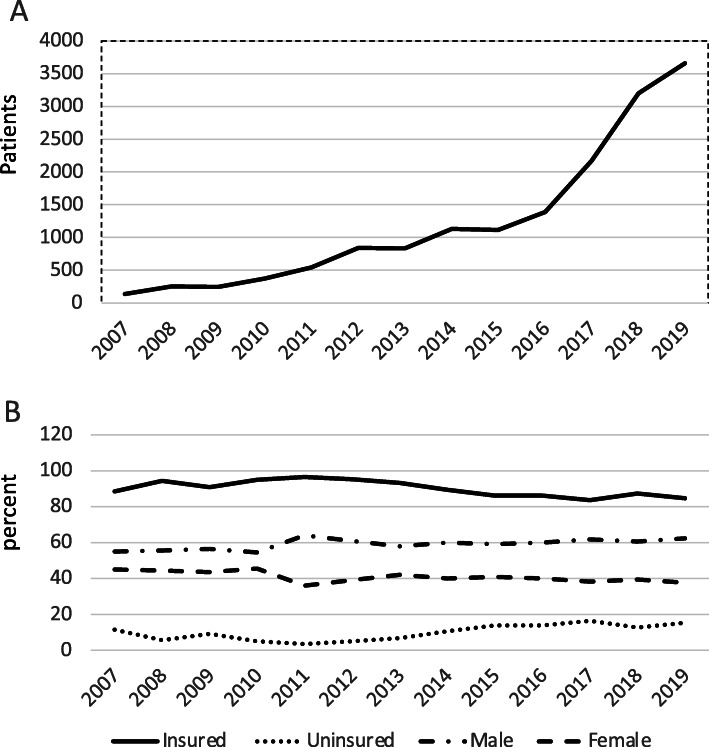
Number of patients wait-listed for kidney transplant (**a**), and percentage of incident patients who were wait-listed, by gender and insurance status (**b**)

The annual number of listed patients increased significantly in the study period, from 138 cases in 2007 to 3, 660 patients in 2019 (Fig. 4a), mainly among the insured population (87.6%). Although the proportion of uninsured patients listed increased slightly over the years, it remained significantly low at 12.4%, while the proportion of females remained around 40% (Fig. 4b). The counts and proportions are presented in Table 3.

**Table 3:** Patient counts and patient proportions on the waiting list, by age, gender, and insurance status. 2007-2019

Year	2007 (%)	2008 (%)	2009 (%)	2010 (%)	2011 (%)	2012 (%)	2013 (%)	2014 (%)	2015 (%)	2016 (%)	2017 (%)	2018 (%)	2019 (%)	Total (%)
Age (y)														
3-14	13 (9.4)	12 (4.8)	4 (1.7)	25 (6.6)	12 (2.2)	33 (3.9)	21 (2.5)	8 (0.7)	12 (1.1)	15 (1.1)	20 (0.9)	24 (0.7)	52 (1.4)	251 (1.5)
15-29	58 (42.0)	116 (46.0)	118 (48.8)	173 (45.8)	249 (45.9)	322 (38.1)	295 (35.5)	440 (38.9)	388 (34.7)	464 (33.5)	632 (29.2)	891 (27.8)	991 (27.1)	5137 (32.3)
30-64	67(48.6)	119 (47.2)	118 (48.8)	174 (46.0)	272 (50.2)	476 (56.3)	500 (60.2)	667 (59.0)	698 (62.5)	881 (63.6)	1469 (67.8)	2206 (68.9)	2509 (68.6)	10156 (63.9)
≥ 65	0 (0)	5 (2.0)	2 (0.8)	6 (1.6)	9 (1.7)	14 (1.7)	15 (1.8)	16 (1.4)	19 (1.7)	26 (1.9)	46 (2.1)	80 (2.5)	108 (3.0)	346 (2.1)
Total	138	252	242	378	542	845	831	1131	1117	1386	2167	3201	3660	15890
Gender														
Male	76 (55.1)	140 (55.5)	137 (56.6)	206 (54.5)	347 (64.0)	515 (60.9)	480 (57.8)	680 (60.1)	661 (59.2)	831 (60.0)	1337 (61.7)	1941 (60.6)	2280 (62.3)	9631 (60.6)
Female	62 (44.9)	112 (44.4)	105 (43.4)	172 (45.5)	195 (36.0)	330 39.1)	351 (42.2)	451 (39.9)	456 (40.8)	555 (40.0)	830 (38.3)	1260 (39.4)	1380 (37.7)	6259 (39.4)
Insured														
Yes	122 (88.4)	238 (94.4)	220 (90.9)	359 (95.0)	523 (96.5)	804 (95.1)	774 (93.1)	1011 (89.4)	970 (86.8)	1195 (86.2)	1811 (83.6)	2795 (87.3)	3101 (84.7)	13923 (87.6)
No	16 (11.6)	14 (5.6)	22 (9.1)	19 (5.0)	19 (3.5)	41 (4.9)	57 (6.9)	120 (10.6)	147 (13.2)	191 (13.8)	356 (38.3)	406 (12.7)	559 (15.3)	1967 (12.4)

During the study period 9508 DD transplants were performed, representing 59.8% of the patients listed in the same time period; waiting time was available in 9362 cases. Median waiting time to transplant was 1.55 (IQR 0.56–3.14) years. Waiting time varied significantly by insurance status, health care system, and gender. Uninsured patients spent less time in the wait list, in comparison with patients with health care insurance.

(1.21, IQR 0.44–2.31, years vs 1.95, IQR 0.66–4.13, years, *p* = 0.0001). IMSS patients waited longer, 2.46 (IQR 1.03–4.70) years to get transplanted, in comparison with the Ministry of Health patients (1.21, IQR 0.44–2.30, years, *p* = 0.0001), and with private patients (0.61, IQR 0.20–1.50, years, p = 0.0001). The median waiting time to transplant was shorter in males than in females (1.51 (IQR 0.51–3.04) years vs 1.61 (IQR 0.62–3.31) years, *p* = .001). The highest deceased-donor transplant rates (pmp) were reported in Mexico City (22.4), Guanajuato (13.8), Sonora (13.5), San Luis Potosí (12.9), and Nuevo León (12.1), Table 4.

**Table 4:** Counts and unadjusted transplant rates of deceased-donor kidney transplants, by state

State	*n* = 9508	pmp
Mexico City	2645	22.45
Guanajuato	1051	13.8
Sonora	502	13.5
San Luis Potosí	459	12.9
Nuevo Leon	809	12.1
Jalisco	946	9.24
Aguascalientes	143	8.42
Chihuahua	363	7.7
Baja California Sur	62	6.79
Puebla	496	6.1
Queretaro	160	6.0
Coahuila	262	6.8
Yucatan	146	5.3
Sinaloa	204	5.2
Michoacán	239	3.9
Tlaxcala	45	2.7
Baja California	115	2.65
Veracruz	244	2.3
Mexico state	397	1.87
Hidalgo	70	1.8
Chiapas	11	1.6
Durango	35	1.53
Tamaulipas	58	1.2
Campeche	1	1.1
Morelos	10	0.4
Zacatecas	7	0.3
Quintana Roo	7	0.3
Colima	14	0.15
Nayarit	3	0.1
Guerrero	2	0.0
Oaxaca	1	0.0
Tabasco	1	0.0
MEXICO	9508	6.0

*pmp* per million population

#### Donors

Between 2007 and 2019, there were 30,907 kidney donors, 25,785 (83.4%) LD and 5522 (16.6%) DD. Overall, LD were largely females (53.1% vs 46.9%, p = 0.0001), while males represented the largest proportion (64% vs 36.0%, p = 0.0001) of DD. The largest proportion (49.3%) of DD kidneys were retrieved at the Ministry of Health facilities, followed by IMSS (36.0%), and private facilities (10.7%). The number of DD, aged 1–91 years, with at least one kidney retrieved increased 82.3%, from 272 in 2007 to 496 in 2019. However, deceased-organ donation rates increased modestly from 2.5 pmp in 2007 to 3.9 pmp in 2019. Average annual donation rate was 3.2 pmp (Table 5). The five leading states in deceased-organ donation rates were Mexico City (8.0 pmp), Aguascalientes (7.5 pmp), Guanajuato and Sonora (7.1 pmp, each), and San Luis Potosí (7.0 pmp). Most of the less developed southern states reported rates < 1.0 pmp. (Table 6).

**Table 5:** Deceased donor count, by gender and insurance status, and overall annual organ donation rate

Year	2007	2008	2009	2010	2011	2012	2013	2014	2015	2016	2017	2018	2019
Gender													
Male	171	185	174	174	217	238	272	266	287	316	323	353	301
Female	101	114	106	110	121	150	138	141	156	156	178	179	195
Insured	140	156	143	133	151	168	180	203	231	260	261	279	293
Uninsured	132	143	137	151	187	220	230	204	212	212	240	253	203
Total	272	299	280	284	338	388	410	407	443	472	501	532	496
Rate pmp	2.5	2.7	2.5	2.5	2.9	3.3	3.4	3.3	3.6	3.8	4.0	4.2	3.9

*pmp* per million population

**Table 6:** Counts and unadjusted annual rates (pmp) of deceased-organ donation, by state of origin

State	n	pmp
Mexico City	939	8.0
Aguascalientes	111	7.5
Guanajuato	545	7.1
Sonora	266	7.1
San Luis Potosí	248	7.0
Baja California Sur	56	6.0
Nuevo León	381	5.7
Queretaro	124	4.6
Chihuahua	207	4.4
Coahuila	150	3.9
Sinaloa	146	3.7
Jalisco	346	3.3
Puebla	264	3.2
Yucatán	87	3.1
Zacatecas	65	3.1
Michoacán	184	3.0
Durango	63	2.7
Estado de México	458	2.1
Morelos	47	1.8
Colima	17	1.8
Tlaxcala	29	1.7
Baja California	72	1.6
Veracruz	132	1.2
Hidalgo	46	1.2
Tamaulipas	47	1.0
Nayarit	15	0.9
Quintana Roo	15	0.7
Guerrero	30	0.6
Tabasco	15	0.4
Oaxaca	8	0.1
Chiapas	7	0.1
Campeche	2	0.1
MEXICO	5122	3.2

*pmp* per million population

## Discussion

Our results describe serious disparities in organ donation and access to kidney transplantation in Mexico. Although the introduction of legislation on organ donation and transplantation in 1984 resulted in a significant increase in kidney transplantation in Mexico, this success was not shared equally by the Mexican population. Although access to health care insurance in Mexico has been a constitutional right since 1983 [3], patients without social security must bear the cost of transplant surgery, including the expenses incurred in living-donor and deceased-donor organ retrieval, as well as the cost of maintaining the immunosuppressive therapy [7]; as a result transplantation rates remained significantly lower among the uninsured population (9.3 pmp vs. 41.7 pmp) in comparison with patients with health care insurance. Similarly, uninsured patients are less likely to being registered in the national transplant waiting list. Only 12% of patients listed in the study period belonged to this population.

To correct the inequities in health care, Seguro Popular (Popular Health Insurance) was implemented in 2003 to provide publicly subsidized health insurance to over half of the Mexican population not covered by social security [17]. However, because Seguro Popular never paid for the cost of kidney transplantation and immunosuppression therapy, except in patients < 18 years old from 2014 until present, transplant rates remained significantly low in the population without social security [18]. Similar to other reports [19], transplant uninsured patients, frequently lose their kidney graft because they abandon their immunosuppressive therapy when it becomes unaffordable, and the financial burden imposed by transplantation frequently aggravates their poverty [9, 20]. In 2020, Seguro Popular was cancelled by the government and replaced by a new system under the Institute of Health and Welfare (INSABI by its Spanish acronym) [21]. Although INSABI has pledged to pay for all health services, there is no clear indication whether it will include kidney transplantation, and the future of equal access to kidney transplantation remains uncertain.

Similar to a previous report in the Mexican population [8], we found that transplant rates were significantly lower in women than in men (37.6% vs 62.4%), while females represented the majority of LD (53.1% vs 46.9%). Likewise, less than half of the patients listed on the national transplant list were women. Studies from other countries like Brazil, the US, France, China, and India report similar findings [8, 22–26].

An additional barrier to transplantation is the bureaucratic nature of the Mexican public health care system. As described by Crowley-Matoka, similar to activities outside the health sector, successfully navigating the health care system in Mexico requires the ability to deploy a combination of favors, personal connections, and political pressure, locally described by the verb *agilizar* (to speed up). Patients who don’t have enough economic and intellectual resources to facilitate their access to transplant candidacy, are seldom transplanted [8]. In addition, patients without health insurance must often self-fund their treatment costs, sometimes by selling their personal property or street begging, and must manage a wide range of formal and informal (paper-work) health information [9, 27, 28].

As described a decade ago, kidney transplantation in Mexico continues to be a family affair [8]. Over 60% of all kidney transplants were performed using organs from living-related donors. Although in our study we could not identify the donor-recipient kinship, it is known that Mexican mothers, wives, and sisters are more likely to be donors, a decision probably driven by economic dependency. Additionally, organ donation in Mexico is identified as another form of nurturing, a “women’s work”, leading to what Crowley-Matoka has described as the *domestication* and *feminization* of organ donation in Mexico [8]. Although, less than 10% of kidney transplants were done using organs from LURD, this type of transplantation represented 20% of the transplants performed in private facilities, raising concerns of organ commercialization [8, 29].

Kidney transplantation from DD organs represented 27% of all kidney transplants performed in the study period. Although DD kidney transplantation rates increased from 4.6 pmp in 2007 to 7.2 pmp in 2019, they remained among the lowest rates in Latin America, in comparison with Uruguay (42.3 pmp), Argentina (24.6 pmp), and Brazil (23.4 pmp) [11]. As expected, deceased organ donation rates (3.2 pmp) are also among the lowest in Latin America, only above low-income countries like Peru (2.0 pmp) Bolivia (0.4 pmp), and Guatemala (0.3 pmp) [11]. The cause of these lower rate is multifactorial. Although the shortage of deceased organ donors in Mexico has traditionally been attributed to public ignorance about organ donation and of family refusals to donate, this perception seems to be inaccurate. In a small prospective report of 64 potential deceased donors, in which 42 families were asked to donate, nine (21.4%) were lost to family refusal to donate, and 21 families consented to donate, for an overall consent rate of 50% [8], similar to the estimated rate reported in the US [30]. Furthermore, in two surveys on organ donation in Mexico, 80% of the interviewed persons knew about organ donation, and 75 to 89% would authorize organ donation from a deceased relative [31, 32].

Because of Mexico’s multi-tiered health care system, deceased-organ retrieval and allocation is also fragmented. In addition to CENATRA, there are 32 state coordinating centers and 553 programs (each with their own waiting list) licensed to practice organ and tissue transplantation [6]. The result is an unequal and inefficient DD organ procurement and distribution that has been described in detail elsewhere [4]. Briefly, although the national coordinating center oversees organ donation and transplantation, in practice every state independently coordinates the local logistics of organ procurement and distribution [33]. There is a national waiting list. However, each transplant facility manages its own waiting list. Procured organs are first offered to patients listed in the hospital where donation has occurred. If no suitable recipients are found, organs are offered to other facilities within the same health system (Social Security, Ministry of Health, or private facilities). Finally, if no candidate is eligible in the same health system or within the state, the organ is offered to any institution with a suitable recipient.

This scheme has led to substantial discrepancies in access to DD transplantation and has increased the gap between the insured and uninsured populations. Patients listed in public health care systems with large waiting lists, like IMSS, must wait longer than those listed in the Ministry of Health and private facilities, where wait lists are significantly smaller. To circumvent this barrier, IMSS or ISSSTE patients who can afford private transplantation or who have the skills to negotiate their listing in the Ministry of Health facilities, “migrate” to these programs where they can get a kidney transplant faster [34]. Once transplanted, they return to IMSS or ISSSTE for follow-up care, including immunosuppressive therapy. Unfortunately, uninsured patients, who can’t afford private medicine and are ineligible to access social security facilities, and whose only chance to get transplanted is at the Ministry of Health facilities, must now “compete” for a deceased-donor organ with patients with health care insurance. Furthermore, they frequently decline the organ because of the lack of funds to finance the transplant when called upon for surgery, missing the opportunity to get transplanted [27]. Additionally, there are geographic restrictions. Most dialysis and transplantation facilities are located in the country’s largest cities and the nation’s capital, which is a far distance for the uninsured population [7].

Finally, although the national transplant registry has been in operation for 32 years, reports on patient and graft survival are missing. Reports on outcomes come from single-center reports [4]. Kidney transplantation counts and rates in the state of Jalisco have been published since 2003 in the USRDS’ International Comparisons section. Recently, national data provided by CENATRA was published for the first time in the 2018 USRDS report [10].

Our study has a number of limitations. Because of the lack of CENATRAS’ reports on patient and graft survival, and to the absence of a national dialysis registry, we could not assess the accountability and transparency of kidney transplantation in Mexico. Time spent on the waiting list was estimated from the date of listing to the date of transplant, and not from the date of starting dialysis. Likewise, transplant rates were estimated per million population, and not to the relative size of the prevalent dialysis population. We could not identify the donor-recipient kinship, although it is known that Mexican women are more likely to be donors, a decision probably driven by economic dependency. Finally, since insurance status is not included in the CENATRA database, the uninsured transplantation rates were estimated by the number of patients transplanted at the Ministry of Health facilities. Since a small proportion of insured patients manage to get transplanted in these settings, this could lead to an overestimation of the results among the uninsured transplant population.

Although our results describe serious disparities in organ donation and access to kidney transplantation in Mexico based on insurance status and gender, other factors such as patient’s preferences, transplant literacy, and bureaucratic barriers imposed by the public health care system cannot be ascertained by our study. However, since the reporting of any organ procurement and transplantation is mandatory by federal law, we consider our results as representative of transplant care at the national level.

## Conclusions

In conclusion, we found serious inequities in access to kidney transplantation in Mexico, which remains restricted largely to patients with public or private health insurance. Uninsured patients (over half of the Mexican population) continue to bear the expenses of transplant surgery and immunosuppressive therapy, restricting their opportunities to access transplantation, and increasing the likelihood of financial ruin. Bureaucratic barriers imposed by the public health system, aggravate the access to kidney transplant to those who most need it. An inefficient and unequal deceased organ procurement and allocation system is largely responsible for the lower rates of deceased organ donation and transplantation. Finally, the absence of reports on patient and graft survival, together with the lack of a dialysis registry, raises concerns about the accountability and transparency of kidney transplantation in Mexico.

To correct these problems, Mexico needs a strategy to establish a national comprehensive kidney care program as outlined by the International Society of Nephrology [2]. The extension of INSABI to cover the expenses of preventive interventions to retard or prevent progression of kidney disease and renal replacement therapies, should be the highest priority. Kidney transplantation must be recognized as the therapy of choice for renal failure. The use of generic immunosuppressive drugs could help to make this therapy more affordable. Outcomes on kidney transplantation must be part of the annual report of the transplant registry. Barriers within the health systems must be eliminated by transparency and accountability of the process of care. To avoid inequalities in organ allocation to those in the waiting list, a national dialysis registry is urgently needed, and time on the waiting list must be counted from the time at wait listing to dialysis commencement, similar to what has been implemented in the US and the UK [35]. These interventions offer an opportunity to correct the unacceptable disparities described in the current report.

## Data Availability

The datasets generated and/or analyzed during the current study are available in the Centro Nacional de Trasplantes (CENATRA) website. https://datos.gob.mx/busca/dataset/pacientes-en-espera-de-un-organo-o-tejido-al-31-de-diciembre-de-2019 https://datos.gob.mx/busca/dataset/trasplantes-de-organos-y-tejidos-2007-2019 https://datos.gob.mx/busca/dataset/donaciones-de-organos-y-tejidos-con-fines-de-trasplante-2007-2019
